# A Systematic Review and Meta-Analysis of Retinal Microvascular Features in Alzheimer's Disease

**DOI:** 10.3389/fnagi.2021.683824

**Published:** 2021-06-29

**Authors:** Qifang Jin, Yiming Lei, Ruoxin Wang, Huiying Wu, Kaibao Ji, Ling Ling

**Affiliations:** ^1^Department of Ophthalmology, The Second Affiliated Hospital of Nanchang University, Nanchang, China; ^2^Department of School of Ophthalmology and Optometry, Nanchang University, Nanchang, China; ^3^Department of Ophthalmology, The First Hospital of Xi'an, Xi'an, China; ^4^Nanchang Bright Eye Hospital, Nanchang, China; ^5^Department of Ophthalmology, Renmin Hospital of Wuhan University, Wuhan, China; ^6^Affiliated Eye Hospital of Nanchang University, Nanchang, China

**Keywords:** Alzheimer's disease, retinal, microvasculature, meta-analysis, optical coherence tomography angiography

## Abstract

**Objective:** The aim of this meta-analysis was to investigate retinal microvascular features in patients with Alzheimer's disease (AD) using optical coherence tomography angiography (OCTA).

**Methods:** PubMed, Cochrane Library, Embase, and Web of Science databases were systematically searched for published articles comparing retinal microvascular characteristics in subjects with AD and controls. The mean difference (MD) with a 95% confidence interval (CI) was used to assess continuous variables. Review Manager Version (RevMan) 5.30, was employed to analyze the data.

**Results:** Nine studies were included in the meta-analysis. The analysis revealed that the macular whole enface superficial and deep vessel density (VD) values measured by OCTA were significantly lower in patients with AD than in controls (MD = −1.10, *P* < 0.0001; MD = −1.61, *P* = 0.0001, respectively). The value measured by OCTA for parafoveal superficial VD in patients with AD was also remarkably lower than that in the control group (MD = −1.42, *P* = 0.001), whereas there was no significant difference in the value for parafoveal deep VD (MD = −3.67, *P* = 0.19), compared to the controls. In addition, the foveal avascular zone (FAZ) was larger in patients with AD than in the control group (MD = 0.08, *P* = 0.07), although it did not reach statistical significance.

**Conclusions:** The present meta-analysis indicated that the macular whole enface and parafoveal vessel densities were reduced in patients with AD. Moreover, our pooled data revealed that FAZ is larger in patients with AD. Consequently, OCTA may be utilized as a diagnostic tool to identify and monitor patients with AD.

## Introduction

Alzheimer's disease (AD) is a neurodegenerative disease characterized by a continuous and irreversible decline in cognitive function (Ashraf et al., 2016). The number of people living worldwide with AD is ~47 million, and this number is expected to triple by 2050 (Baumgart et al., 2015). Although the exact mechanism of AD remains unclear, AD is mainly associated with redundant extracellular deposition of β-amyloid (Aβ) plaques, loss of neurons, intracellular accumulation of neurofibrillary tangles, and abnormal presence of the intracellular tau protein (Ballard et al., 2011; Wang et al., 2017). Multiple risk factors contributing to the onset of AD include smoking, depression, obesity, diabetes mellitus, stroke, cerebral atherosclerosis, arteriosclerosis, and hypertension (Barnes and Yaffe, 2011; Lemos et al., 2015). The diagnosis of AD was mainly based on clinical criteria combined with neuroimaging for the detection of the abnormal biomarkers of Aβ deposition (Tang et al., 2020). Cerebrospinal fluid (CSF) or positron emission tomography (PET) was commonly used to detect amyloid beta concentration (Jack et al., 2018). However, these methods are invasive, expensive, and time-consuming (den Haan et al., 2017). Recent studies have shown that reduced cerebral blood flow and blood-brain barrier changes may be involved in the pathogenesis of AD (Iturria-Medina et al., 2016; Sweeney et al., 2018). Another study reported that cerebral hypoperfusion not only increased the cerebral vascular tortuosity but also decreased vascular density in patients with AD (Yamashita et al., 2014). However, the cerebral vasculature is too small to be directly visualized *in vivo* (Smith and Beaudin, 2018).

Retinal and cerebral vessels share similar features (Hughes et al., 2000). A previous study has revealed retinal microvascular alterations in patients with AD using fundus photographs (Cheung et al., 2014). Consequently, an increasing number of researchers are focusing on investigating the retinal vascular characteristics to evaluate the cerebral vasculature (Berisha et al., 2007). Optical coherence tomography angiography (OCTA) is a new and non-invasive angiographic technique that enables visualization and quantification of the retinal and choroidal vessels without intravenous injection of a fluorescein dye (Ling et al., 2020). It is preferable to measure the retinal vasculature at distinct depths, separating the superficial and deep capillary layers. However, whether there are retinal microvasculature alterations in patients with AD is yet to be fully determined. Several studies using OCTA have reported significantly decreased vascular density in the eyes of patients with AD compared to controls (Jiang et al., 2018; Wang et al., 2021), and this change was positively associated with cognitive dysfunction (Yan et al., 2021). In contrast, another study revealed that there were no significant differences in the retinal vascular density between patients with AD and controls (Querques et al., 2019). Given the inconsistent results regarding retinal microvascular characteristics in AD, a comprehensive meta-analysis of existing studies is urgently needed. To our knowledge, no meta-analysis has comprehensively assessed the retinal microvascular features observed in patients with AD.

Therefore, we performed this meta-analysis in an effort to evaluate retinal microvascular features in patients with AD and provide more reliable evidence for the utility of OCTA in the diagnosis and monitoring of patients with AD.

## Materials and Methods

### Literature Search Strategy

This study was performed in accordance with the Preferred Reporting Items for Systematic Reviews and Meta-analysis (PRISMA) guidelines (Moher et al., 2009). As previously published articles were screened, no ethical approval was required. Two (Qifang Jin and Kaibao Ji) independent authors comprehensively searched all published studies in electronic databases, including PubMed, Embase, Cochrane Library, and Web of Science from inception through February 24, 2021. The following search terms were used: “Alzheimer disease,” “dementia,” “optical coherence tomography angiography,” “optical coherence tomographic angiography,” “OCTA,” and “OCT angiography.” The eligible studies were limited to those in the English language and conducted in human subjects.

### Inclusion and Exclusion Criteria

The eligible criteria adhered to PICOS (population, intervention, control, outcome, and study design) principle. Included studies had to satisfy the following criteria: (1) AD patients met the diagnostic criteria of the National Institute of Neurological and Communicative Disorders and Stroke/Alzheimer's Disease and Related Disorders Association criteria (McKhann et al., 1984), and confirmed by β amyloid deposition using cerebrospinal fluid (CSF) or positron emission tomography (PET) (Jack et al., 2018); (2) studies comparing retinal microvasculature features in patients with AD with healthy controls using OCTA; (3) individuals with normal cognition served as healthy controls; (4) primary outcomes included macular whole enface superficial and deep vessel density (VD), parafoveal superficial and deep VD, and foveal avascular zone (FAZ); original data should contain the mean ± standard deviation (SD); (5) study design was case-control study.

Studies were excluded if: (1) they were duplicate publications; (2) they were case reports, animal studies, abstracts of conferences, reviews, comments, and letters to the editor; (3) they reported inadequate outcomes or outcomes could not be extracted; and (4) study purpose did not meet the inclusion criteria.

### Data Extraction and Quality Evaluation

Two authors (Qifang Jin and Kaibao Ji) independently collected the data from the included studies, and any disagreements were resolved through discussion. Data including first author, country, publication time, study design, sample size, mean age, OCTA device, outcomes, and quality scores were collected from each study. The quality of case-control studies was assessed using the Newcastle-Ottawa Scale criteria, which provides a scale range of 0–9 points, and studies with scores ≥ 5 were regarded as high quality (Stang, 2010).

### Statistical Analysis

Review Manager Version 5.30 (Cochrane Collaboration, Oxford, UK) software was adopted to perform statistical analysis. Continuous variables were assessed in terms of mean difference (MD) with 95% confidence interval (CI). The heterogeneity among studies was analyzed using the Chi-squared test based on the values of *I*^2^ and *P*. *I*^2^ statistic ranges from 0 to 100%, with 25, 50, and 75% indicating low, moderate, and high heterogeneity, respectively. A fixed-effect model was adopted when little heterogeneity among studies; otherwise, a random-effect model was employed. *P* < 0.05 was indicated significant difference.

## Results

### Search Results

**Figure 1:**
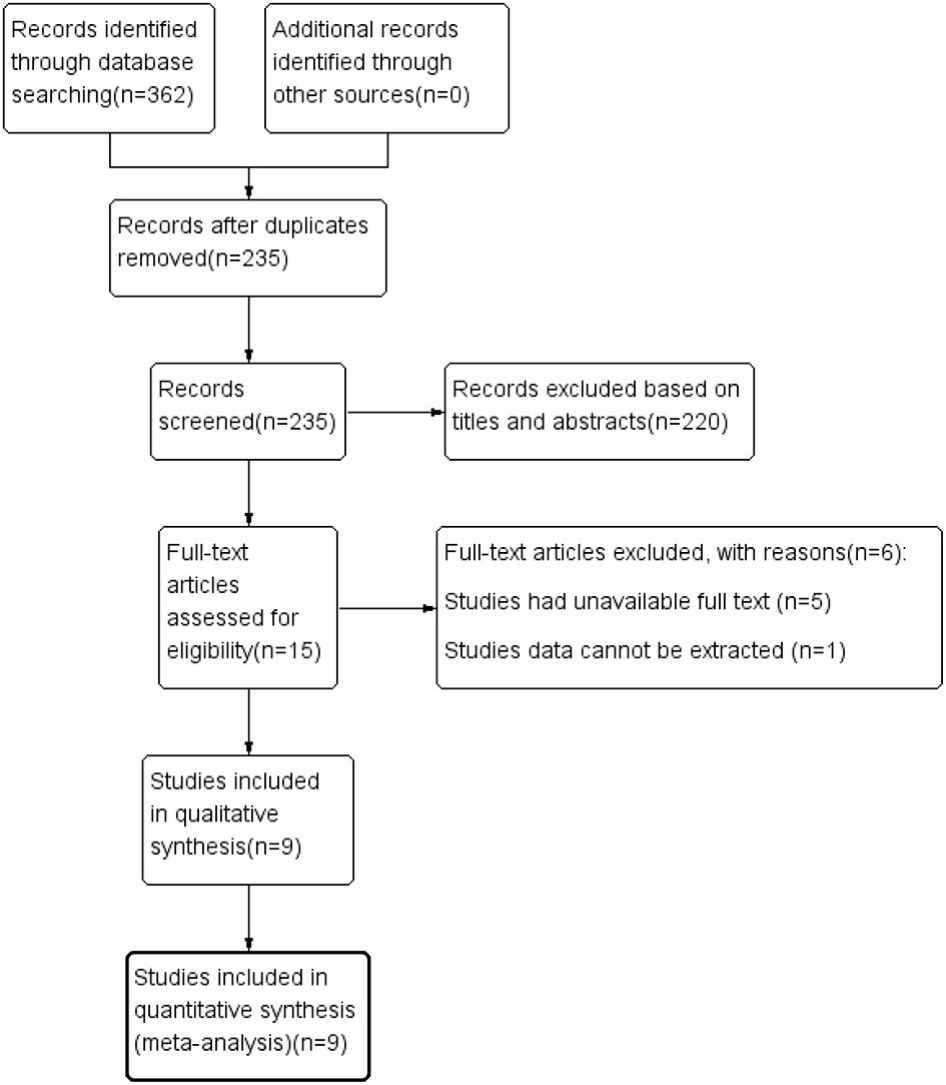
Flow diagram by which eligible articles were selected.

The literature screening process is illustrated in Figure 1. A total of 362 studies were preliminarily retrieved from all databases (PubMed: 117, Embase: 178, Web of Science: 66, Cochrane Library: 1), of which 127 were excluded due to duplication. We subsequently removed 220 unrelated articles after reviewing the titles and abstracts. After the remaining 15 potential articles were reviewed in full text, six articles were excluded as five studies had unavailable full text while data of one study could not be extracted. Finally, nine eligible articles (Bulut et al., 2018; Lahme et al., 2018; den Haan et al., 2019; Querques et al., 2019; Yoon et al., 2019; Zabel et al., 2019; Chua et al., 2020; Wu et al., 2020; Wang et al., 2021), with a total of 859 eyes (333 in the AD group and 526 in the control group) were included in this meta-analysis.

Table 1 displays the general characteristics of the nine selected studies. Table 2 demonstrates the results of quality assessment of the included articles.

**Table 1:** General characteristics of the eligible studies.

**References**	**Place**	**Age (years)**	**Design**	**Number of eyes**	**Gender (M/F)**	**OCTA type**	**Scan size (mm^**2**^)**	**Primary outcomes**	**Clinical diagnostic criteria**
Wang et al. (2021)	China	71.81 ± 7.98 69.50 ± 5.94	Case-control study	AD Cases: 62Controls: 49	27/35 17/32	Optovue	3 ×3 in macular	Foveal whole enface superficial VD, Foveal whole enface deep VD, Parafoveal superficial VD, Parafoveal deep VD, FAZ	NIA/AA
Querques et al. (2019)	Italy	72.9 ± 7.2 71.6 ± 5.9	Prospective Case-control study	AD Cases: 12Controls:32	4/8 17/15	Zeiss	3 × 3/6 × 6 in macular	Foveal whole enface superficial VD, Foveal whole enface deep VD	NIA/AA
Bulut et al. (2018)	Turkey	74.23 ± 7.55 72.58 ± 6.28	Case-control study	AD Cases: 26Controls: 26	11/15 13/13	Optovue	6 × 6 in macular	Foveal whole enface superficial VD, Foveal whole enface deep VD, FAZ	NIA/AA DSM-IV
Lahme et al. (2018)	Germany	67.97 ± 9.30 66.08 ± 10.11	Case-control study	AD Cases: 36Controls: 38	21/15 23/14	Optovue	3 × 3/6 × 6 in macular	Foveal whole enface superficial VD, Foveal whole enface deep VD, Parafoveal superficial VD, Parafoveal deep VD	NIA/AA
den Haan et al. (2019)	Netherland	65.4 ± 8.1 60.6 ± 5.0	Cross-sectional study	AD Cases: 48Controls: 38	25/23 24/14	Zeiss	6 × 6 in macular	Parafoveal superficial VD, FAZ	NIA/AA
Yoon et al. (2019)	USA	72.8 ± 7.7 69.2 ± 7.8	Cross-sectional study	AD Cases: 70Controls: 254	13/26 36/97	Zeiss	3 × 3/6 × 6 in macular	Foveal whole enface superficial VD, Parafoveal superficial VD, FAZ	NIA/AA
Zabel et al. (2019)	Poland	74.11 ± 5.87 74.26 ± 7.66	Cross-sectional study	AD Cases: 27Controls: 27	6/21 8/19	Optovue	3 × 3 in macular	Foveal whole enface superficial VD, Foveal whole enface deep VD, FAZ	NIA/AA DSM-IV
Wu et al. (2020)	China	69.94 ± 6.39 68.67 ± 5.85	Prospective case-control study	AD Cases: 28Controls: 33	10/9 11/10	Optovue	6 × 6 in macular	Paraoveal superficial VD, Paraoveal deep VD, FAZ	NINCDS- ADRDA
Chua et al. (2020)	Singapore	74.9 ± 6.0 76.7 ± 5.3	Case-control study	AD Cases: 24Controls: 29	7/17 16/13	Zeiss	3 × 3 in macular	Foveal whole enface superficial VD, Foveal whole enface deep VD	DSM-IV

*VD, vessel density; FAZ, Foveal avascular zone; NIA/AA, National Institute of Aging-Alzheimer's Association workgroups; DSM-IV, the 4th edition of the Diagnostic and Statistical Manual of Mental Disorders; NINCDS-ADRDA, the National Institute of Neurological and Communicative Disorders and Stroke and Alzheimer's Disease and Related Disorders Association*.

**Table 2:** NOS for evaluating included studies quality.

**Methodological item for non-randomized studies (No. 1-8)**	**Wang et al. (2021)**	**Querques et al. (2019)**	**Bulut et al. (2018)**	**Lahme et al. (2018)**	**den Haan et al. (2019)**	**Yoon et al. (2019)**	**Zabel et al. (2019)**	**Wu et al. (2020)**	**Chua et al. (2020)**
1. Is the case definition adequate?	1	1	1	1	1	1	1	1	1
2. Representativeness of the cases	1	1	1	1	1	1	1	1	1
3. Selection of controls	0	0	0	0	0	0	0	0	0
4. Definition of controls	1	1	1	0	1	1	1	0	0
5. Comparability of cases and controls on the basis of the design or analysis	2	2	2	2	1	1	2	2	2
6. Ascertainment of exposure	1	1	1	1	1	1	1	1	1
7. Same method of ascertainment for cases and controls	1	1	1	1	1	1	1	1	1
8. Non-response rate	0	0	0	0	0	0	0	0	0
Total score	7	7	7	6	6	6	7	6	6

*NOS, Newcastle-Ottawa scale. The selection area included Nos. 1–4, which was up to one score in one question; The comparability area included No. 5, which was up to 2 scores in the question; The exposure area included Nos. 6–8, which was up to one score in one question. The total score was 9*.

### Meta-Analysis Results

#### Macular Whole Enface Vessel Density in Patients With AD and Controls

**Figure 2:**
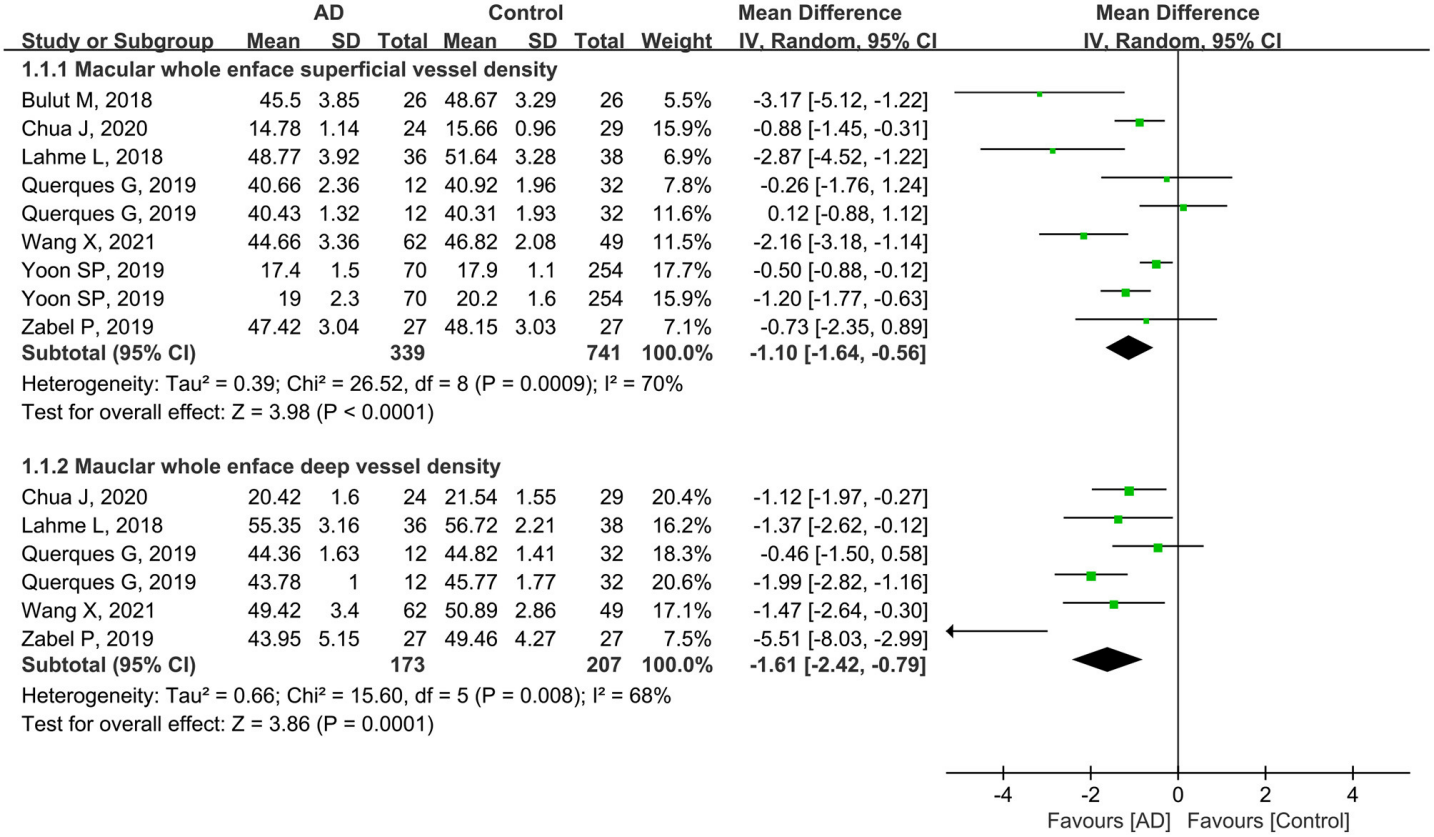
Forest plot of macular whole enface superficial and deep vessel density between AD and control groups.

Seven studies analyzed the macular whole enface superficial VD. The pooled MD was −1.10 (95% CI: −1.64 to −0.56, *P* < 0.0001, Figure 2), revealing that the macular whole enface superficial VD was remarkably lower in patients with AD compared to controls. In five studies that reported macular whole enface deep VD, the pooled MD was also significantly lower in the AD group (MD: −1.61, 95% CI: −2.42 to −0.79, *P* = 0.0001, Figure 2). Although substantial differences were found between the two groups, there was moderate heterogeneity across the studies (chi^2^ = 26.52, *P* = 0.0009, *I*^2^ = 70%; chi^2^ = 15.60, *P* = 0.008, *I*^2^ = 68%; Figure 2).

**Figure 3:**
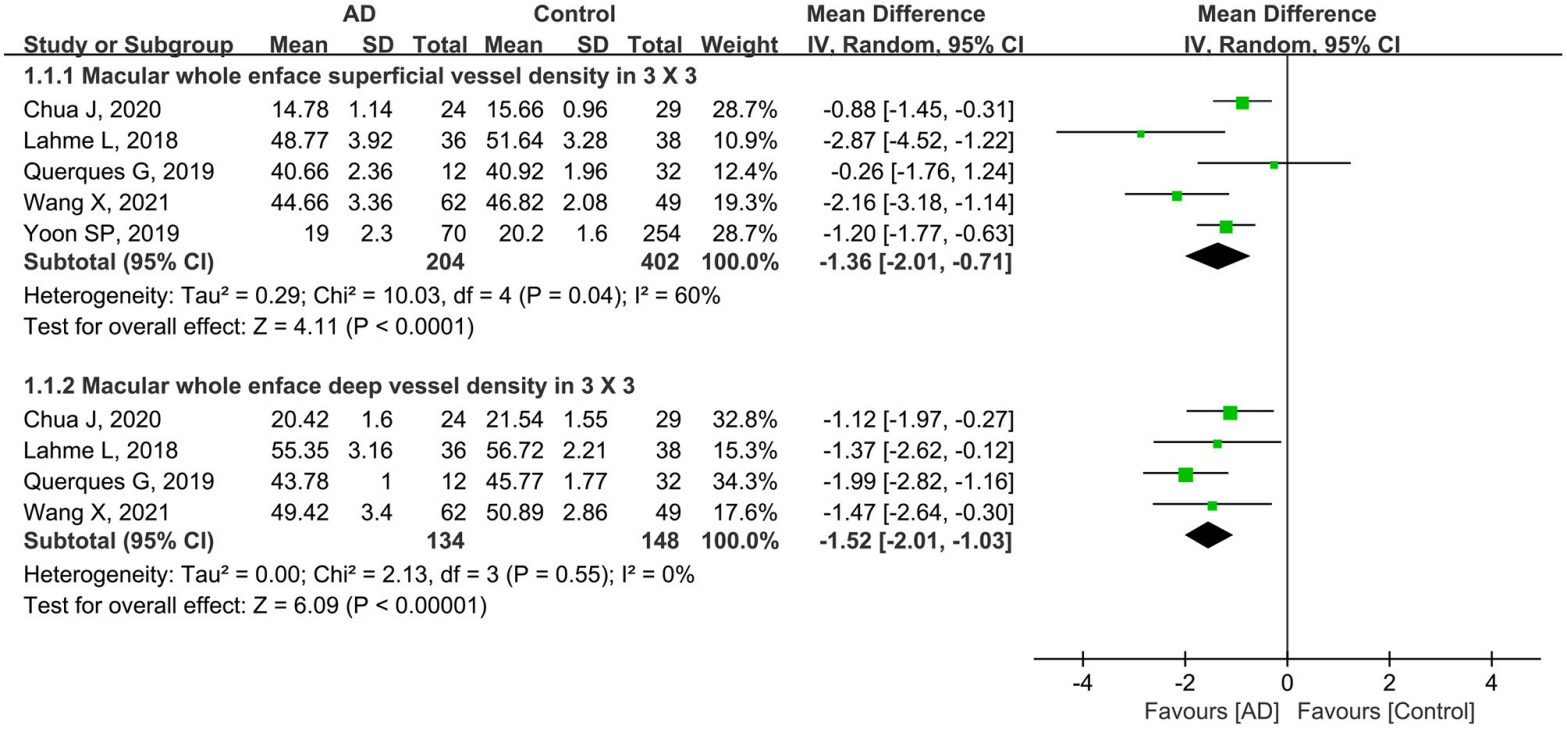
Forest plot of macular whole enface superficial and deep vessel density using a 3 × 3-mm scan size in AD patients and controls.

In addition, five studies including 606 eyes (204 eyes in AD group and 402 eyes in controls) reported the macular whole enface superficial and deep vessel density using a 3 × 3-mm scan size. The pooled MD was −1.36 (95% CI: −2.01 to −0.71, *P* < 0.0001, Figure 3), indicating that the macular whole enface superficial VD was significantly lower in the AD group. However, there was moderate heterogeneity across the studies (chi^2^ = 10.03, *P* = 0.04, *I*^2^ = 60%, Figure 3). Four other studies assessed 282 eyes (134 in AD and 148 in control groups), and analyzed the macular whole enface deep VD using a 3 × 3-mm scan size. Overall, considerable differences in the macular whole enface deep VD using a 3 × 3-mm scan size were found between patients with AD and controls (MD: −1.52, 95% CI: −2.01 to −1.03, *P* < 0.00001, Figure 3), with no heterogeneity across the studies (chi^2^ = 2.13, *P* = 0.55, *I*^2^ = 0%, Figure 3).

**Figure 4:**
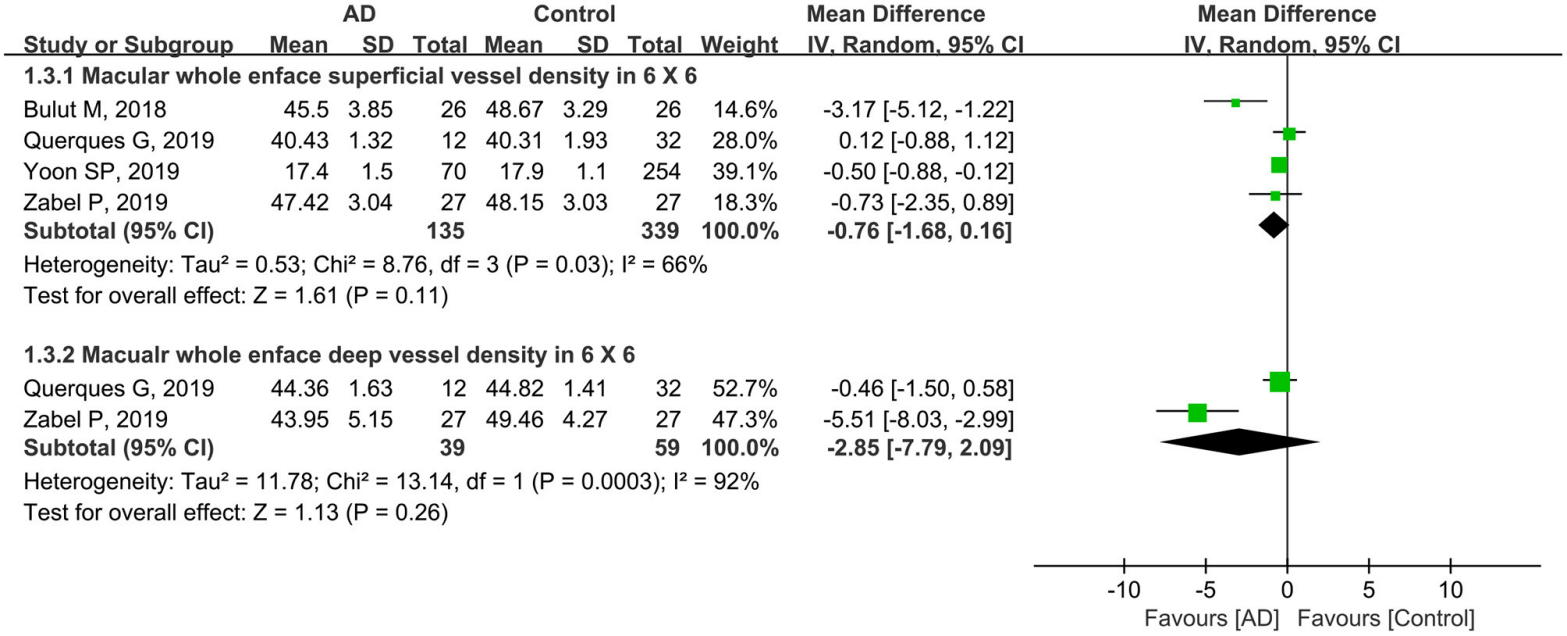
Forest plot for macular whole enface superficial and deep vessel density within a 6 × 6-mm scan size between eyes with AD and the controls.

Furthermore, four studies with a total of 474 eyes (135 eyes in patients with AD and 339 eyes in controls) reported the macular whole enface superficial and deep VD using a 6 × 6-mm scan size. The summary MD in the macular whole enface superficial VD using a 6 × 6-mm scan between the two groups was −0.76 (95% CI: −1.68 to 0.16, *P* = 0.11, Figure 4), demonstrating that the VD was decreased in patients with AD but not significantly. There was moderate heterogeneity across the studies (chi^2^ = 8.76, *P* = 0.03, *I*^2^ = 66%, Figure 4). The subgroup analysis results also showed that the pooled MD in the macular whole enface deep VD as measured using a 6 × 6-mm scan size was lower in patients with AD (MD: −2.85, 95% CI: −7.79 to 2.09, *P* = 0.26, Figure 4), and associated with high heterogeneity across studies (chi^2^ = 13.14, *P* = 0.0003, *I*^2^ = 92%, Figure 4).

#### Parafoveal Vessel Density in Patients With AD and Controls

**Figure 5:**
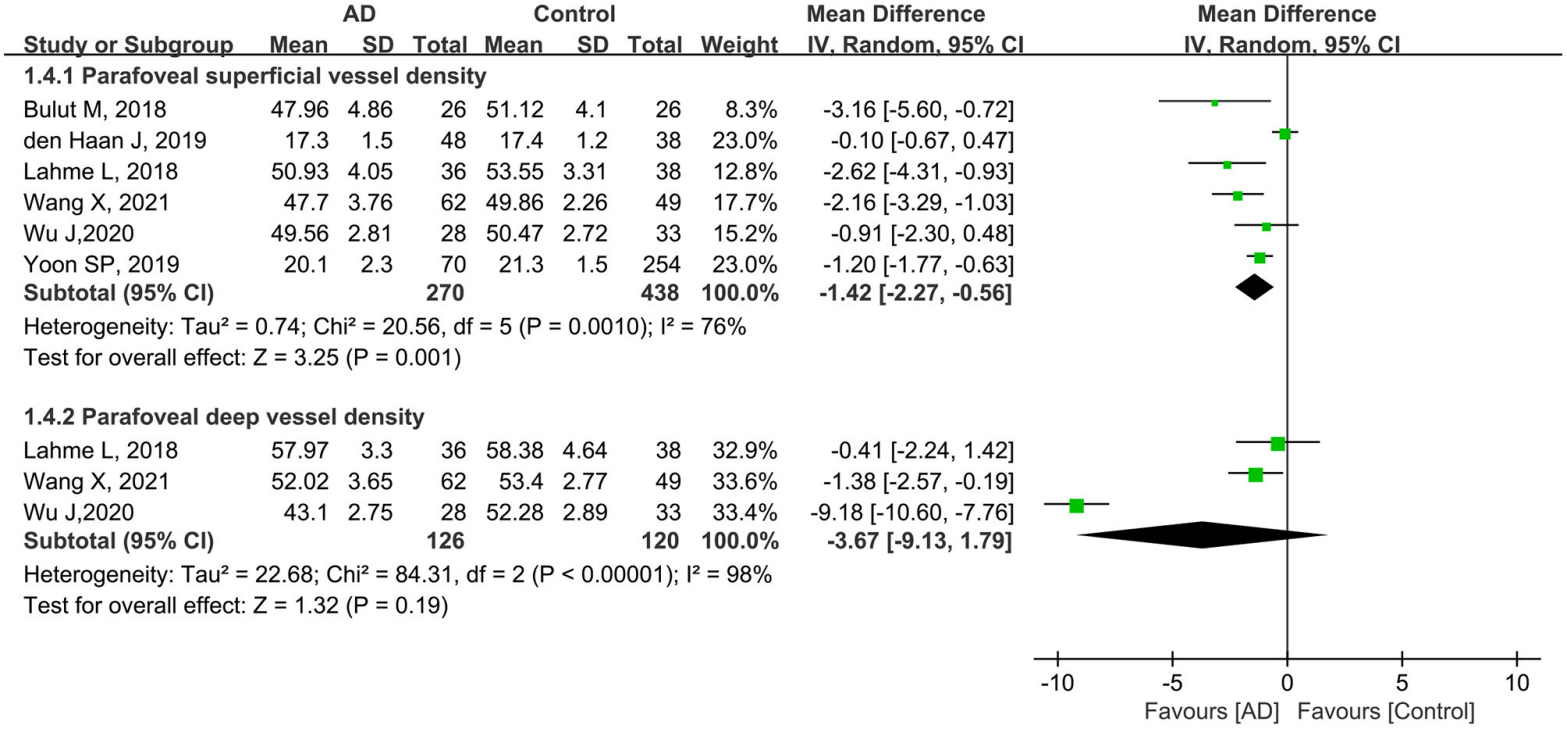
Forest plot of parafoveal superficial and deep vessel density in AD patients and controls.

A total of 708 eyes (270 eyes in the AD group and 438 eyes in the control group) in six studies were included in the analysis of the parafoveal superficial vessel density. Compared with controls, the pooled parafoveal superficial VD (MD: −1.42, 95% CI: −2.27 to −0.56, *P* = 0.001, Figure 5) was significantly lower in the AD group, and associated with high heterogeneity across studies (chi^2^ = 20.56, *P* = 0.001, *I*^2^ = 76%, Figure 5). Three studies reported parafoveal deep vessel density in 126 eyes with AD and 120 control eyes. The pooled MD for parafoveal deep VD revealed high heterogeneity (chi^2^ = 84.31, *P* < 0.00001, *I*^2^ = 98%, Figure 5), and was decreased in eyes with AD, although not remarkably different (MD: −3.67, 95% CI: −9.13 to 1.79, *P* = 0.19, Figure 5).

**Figure 6:**
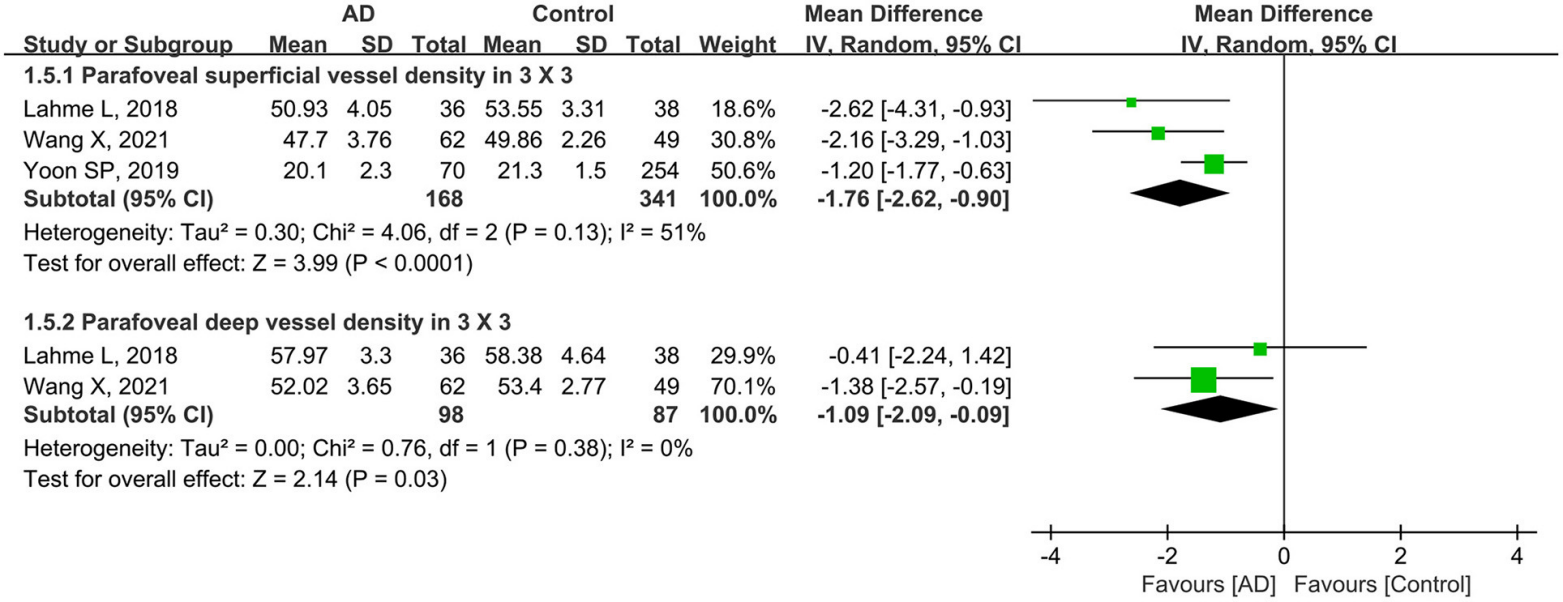
Forest plot for parafoveal superficial and deep vessel density as measured by 3 × 3-mm scan size in AD eyes and controls.

Subgroup analysis of scan size showed that the pooled MD for parafoveal superficial vessel density as measured in a 3 × 3-mm scan size was −1.76 (95% CI: −2.62 to −0.90, *P* < 0.0001, Figure 6), revealing that this parameter was significantly lower in patients with AD with mild heterogeneity across studies (chi^2^ = 4.06, *P* = 0.13, *I*^2^ = 51%, Figure 6). In addition, the parafoveal deep VD (3 × 3-mm scan) was also substantially reduced in eyes with AD (MD: −1.09, *P* = 0.03, Figure 6), with the studies showing minimal heterogeneity (chi^2^ = 0.76, *P* = 0.38, *I*^2^ = 0%, Figure 6).

**Figure 7:**
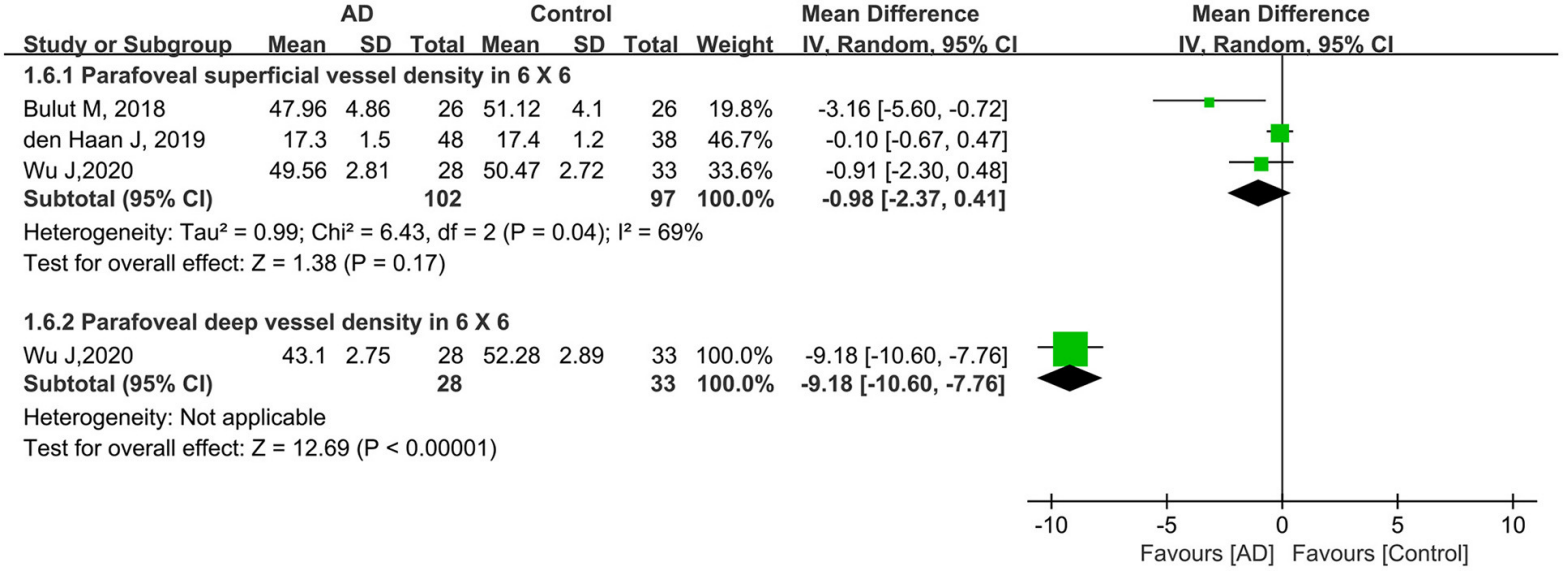
Forest plot for parafoveal superficial and deep vessel density in 6 × 6-mm scan size between AD and control groups.

Furthermore, three other studies analyzed parafoveal superficial VD using a 6 × 6-mm scan in 199 eyes (102 eyes in patients with AD and 97 eyes in controls). Compared to controls, this parameter was lower in eyes with AD (MD: −0.98, 95% CI: −2.37 to 0.41, *P* = 0.17, Figure 7) with moderate heterogeneity across the studies (chi^2^ = 6.43, *P* = 0.04, *I*^2^ = 69%, Figure 7). Only one eligible study reported parafoveal deep VD using a 6 × 6-mm scan in 61 eyes (28 eyes in patients with AD and 33 eyes in controls), and the pooled MD was −9.18 (95% CI: −10.60 to −7.76, *P* < 0.00001, Figure 7), revealing that the parafoveal deep VD (6 × 6-mm scan) was remarkably lower in the AD group.

#### FAZ Analysis in Patients With AD and Controls

**Figure 8:**
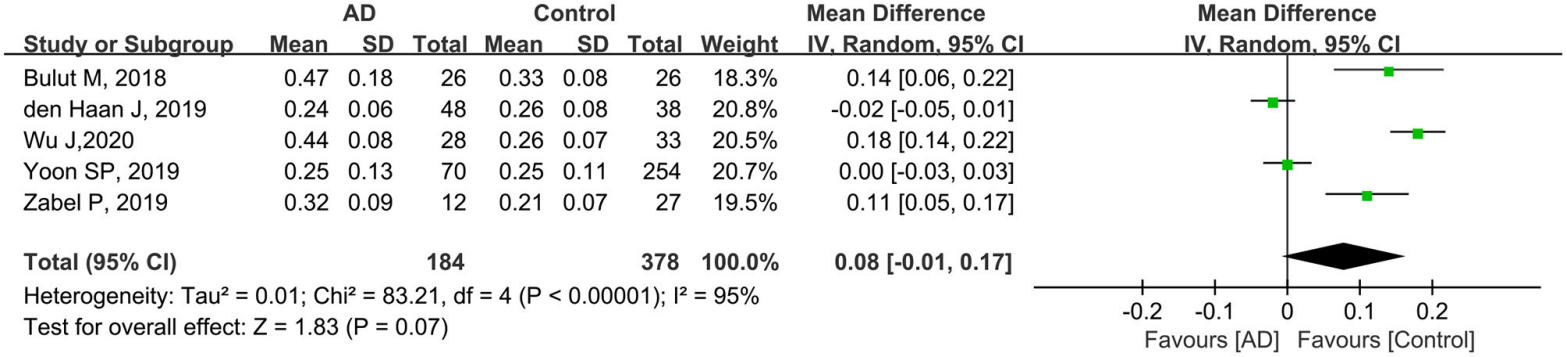
Forest plot for analysis of foveal avascular zone in eyes with AD and controls.

Five studies including 562 eyes (184 eyes in patients with AD and 378 eyes in controls) were assessed to analyze the FAZ. Among these studies, the FAZ was larger in patients with AD, although the difference was not statistically significant (MD: 0.08, 95% CI: −0.01 to 0.17, *P* = 0.07, Figure 8). High heterogeneity was found among these studies (chi^2^ = 83.21, *P* < 0.00001, *I*^2^ = 95%, Figure 8).

## Discussion

To the best of our knowledge, this is the first meta-analysis to evaluate and compare retinal microvasculature alterations using OCTA between subjects with AD and controls. Nine eligible studies, including 859 eyes, were used to assess the differences in the retinal microvasculature between patients with AD and controls. In this comprehensive meta-analysis, we pooled several parameters relevant for diagnosing AD, including macular whole enface superficial and deep vessel density, parafoveal superficial and deep vessel density, and FAZ. Although the heterogeneity across the eligible studies concurred with previous evidence, the pooled data showed that macular whole enface and parafoveal vessel densities were significantly lower in patients with AD. Furthermore, our findings revealed that FAZ was slightly larger in patients with AD.

The pathological hallmark of AD is characterized by redundant extracellular deposition of β-amyloid (Aβ) plaques, loss of neurons, intracellular accumulation of neurofibrillary tangles, and abnormal presence of the intracellular tau protein (Ballard et al., 2011; Wang et al., 2017). Recent studies have shown that reduced cerebral blood flow and blood-brain barrier changes may be involved in the pathogenesis of AD (Iturria-Medina et al., 2016; Sweeney et al., 2018, 2019). Currently, the diagnosis of AD mainly relies on either positron emission imaging or cerebrospinal fluid analysis, which is very expensive and invasive (Florek et al., 2018; Mounsey and Zeitler, 2018). However, the cerebral microvasculature is too small to be directly visualized *in vivo*. Due to the shared embryologic origin of the retina and brain, there is resemblance between blood-retinal barrier (BRB) and blood-brain barrier (BBB), and the retina is thus regarded as an extension of the CNS (London et al., 2013; Gupta et al., 2020; Rifai et al., 2021); retinal vascular lesions, including reduced arterial diameter, expanded venous diameter, and decreased fractional dimension, were found in patients with AD using fundus photographs (Cheung et al., 2014; McGrory et al., 2016). Moreover, accumulating evidence have reported that Aβ deposits were discovered in retinas of AD and animal studies (Hart et al., 2016; Doustar et al., 2017; den Haan et al., 2018). Furthermore, there are accumulating literatures showing that retinal microvasculature serves an accurate window to evaluate cerebrovascular disorders, including stroke, Parkinson's disease (PD), AD, and multiple sclerosis (MS) (Cheung et al., 2017; Bulut et al., 2018; Kwapong et al., 2018; Feucht et al., 2019).

Thus, an increasing number of researchers have explored the retinal vasculature to identify biomarkers to objectively evaluate patients with AD. With the advancement in retinal imaging approaches, OCTA has provided a potential approach for researchers to explore the pathogenesis and progression of AD by quantitatively assessing the retinal vascular features. Previous studies have reported significantly reduced macular VD in patients with AD (Lahme et al., 2018; Zhang et al., 2019; Wu et al., 2020). The findings of our meta-analysis strongly support previous evidence. There is accumulating clinical evidence showing that there is a range of retinal alterations in AD, including constricted retinal vessels with increased tortuosity, atherosclerosis, amyloid angiopathy, and reduced arterial fractal dimensions (Lesage et al., 2009; Golzan et al., 2017; Gupta et al., 2020). The reduced retinal vessels can be explained by faulty Aβ clearance from the retina, as well as abnormal Aβ aggregation around the vascular walls in the retina (Cerquera-Jaramillo et al., 2018; Chua et al., 2020). Excessive Aβ deposition disrupts the normal functioning of the neurovascular unit, with worsening of the regulation of local blood flow due to loss of vascular contractibility, and partial occlusion of the smaller distal arterioles by Aβ aggregates, leading to a deficit in oxygen and blood flow rate (Banerjee et al., 2017; Salobrar-Garcia et al., 2020). In addition, increased apoptosis of the retinal pericytes was found in AD retinas concomitantly with downregulated vascular low-density lipoprotein receptor-related protein 1 (LRP-1), leading to reduced Aβ clearance (Gupta et al., 2020). This was supported by the observation that the accumulation of Aβ in the retina has been closely linked to early loss of pericytes (Carare et al., 2020). These changes have been associated with reduced vascular density.

In our study, subgroup analysis of scan size showed significantly decreased VD in both macular and parafoveal superficial and deep layers as measured by a 3 × 3-mm scan (Figures 3, 6). However, the pooled MD in the macular and parafoveal superficial and deep layers using a 6 × 6-mm scan size was decreased in patients with AD, although not significantly, with moderate heterogeneity among the studies (Figures 4, 7). This noteworthy phenomenon may be attributed to projection artifacts, which are caused by superficial vessels projecting shadows onto the deeper layers of the retina, affecting the results (Zhang et al., 2015; Zabel et al., 2019). Despite using the latest version software equipped with the AngioVue 3D PAR algorithm, the ability to remove projection artifacts in the 6 × 6-mm scan size is <3 × 3-mm scan size (Zabel et al., 2019). Another potential factor was the relatively small number of subjects in the subgroup using the 6 × 6-mm scan size. We suggest that macular whole enface and parafoveal vessel densities in patients with AD should be analyzed in the 3 × 3-mm scan area in future studies.

The reduction in the macular blood vessels may consequently elicit a larger foveal avascular zone. The previous authors found dropout of vasculature specifically within the fovea, leading to enlarged FAZ in the biomarker-positive group (O'Bryhim et al., 2018). The authors also demonstrated that enlarged FAZ could be associated with reduced angiogenesis caused by VEGF being bound and blocked by Aβ (Bulut et al., 2018). In addition, the accumulation of Aβ aggregates in the internal vessel walls results in the occlusion of the vascular structure and a reduction in the blood flow (Berisha et al., 2007; Dorr et al., 2012). Previous studies have shown a significantly enlarged FAZ in subjects with AD compared with controls (Bulut et al., 2018; Zabel et al., 2019; Chua et al., 2020). In our study, we found a slightly insignificant enlargement in the FAZ area in patients with AD compared to the controls (MD: 0.08, *P* = 0.07). We speculate that some factors may have potentially led to this result. First, the relatively small sample size limited the power for assessing this metric between patients with AD and controls. Furthermore, physiological variability of the FAZ area in individuals has been reported in previous studies (Fujiwara et al., 2017; Sampson et al., 2017). Manual delineation of the FAZ region by researchers as well as different segmentation methods for FAZ measurement may also contribute to a bias (Linderman et al., 2017; Chua et al., 2020). To verify our findings, future longitudinal studies with larger sample sizes should be conducted.

There are several noteworthy limitations to our study. First and most importantly, the sample sizes of the included studies were relatively small. Second, the pooled results should be interpreted with caution because statistical heterogeneity existed across the studies. Third, the source of heterogeneity could not be fully identified due to insufficient data to perform a meta-regression. Finally, although our study's protocol was not registered in the PROSPERO database, we found no corresponding registrations in the database. To validate our results, prospective longitudinal studies with sufficient sample sizes will be necessary to evaluate retinal vasculature in patients with AD in the future.

## Conclusion

In the current meta-analysis, we concluded that macular whole enface and parafoveal vessel densities were decreased in patients with AD. In addition, our pooled data revealed that FAZ was larger in patients with AD. Consequently, OCTA may have the potential to detect retinal microvascular deficits in patients with AD.

## Data Availability Statement

The original contributions presented in the study are included in the article/supplementary material, further inquiries can be directed to the corresponding author/s.

## Author Contributions

LL, KJ, and QJ conceived and designed the study. QJ and KJ searched the literature and contributed to writing of original manuscript. YL and RW contributed to data acquisition and analysis. YL and HW were responsible for the software. LL and KJ were responsible for revising and reviewing. All authors contributed to the article and approved the submitted version.

## Conflict of Interest

The authors declare that the research was conducted in the absence of any commercial or financial relationships that could be construed as a potential conflict of interest.
